# VALIDATION OF A BEHAVIOURAL, SPEECH SPECTRUM/SPEECH BASED mHEALTH HEARING SCREENER FOR CHILDREN

**DOI:** 10.12688/wellcomeopenres.25773.1

**Published:** 2026-02-12

**Authors:** Deepashree Joshi, Vidya Ramkumar, Ramya Vaidyanath, Anil Prabhakar

**Affiliations:** 1 Faculty of Audiology and Speech Language Pathology, Sree Balaji Medical College & Hospital, Chennai, Tamil Nadu, 600044, India; 2 Faculty of Audiology and Speech Language Pathology, Sri Ramachandra Institute of Higher Education and Research (Deemed to be University), Chennai, Tamil Nadu, 600116, India; 3 Former Faculty at Department of Audiology, Sri Ramachandra Institute of Higher Education and Research (Deemed to be University), Chennai, Tamil Nadu, 600116, India; 4 Electrical and Electronic Engineering, Indian Institute of Technology Madras Department of Electrical Engineering, Chennai, Tamil Nadu, 600036, India

**Keywords:** mHealth, Hearing, Screening, Children, Pediatric, Validation, community-based, grassroots workers, community workers, Low Middle Income Countries Speech awareness, speech recognition

## Abstract

An affordable mHealth-based subjective hearing screener (SRESHT) for children was developed to support contextualized hearing screening for developing countries. The screener was developed with calibrated commercial headphones and speakers at 60 dB HL with the target goal of identifying children with moderately severe or above degrees of disability (WH0, 2019). The hearing screener includes Behavioral Observation Audiometry (BOA) based screening module (0 to 1 year of age), Speech Spectrum Awareness Task (SSAT) module (1 to 3 years of age), Speech Recognition Task (SRT) module (3 to 6 years of age) and High Risk register (HRR) and Parental Questionnaire (PQ) module (0 to 6 years).

The study identified optimal screening modules for detecting hearing loss = 60 dB HL across different age groups. For infants aged 0–1 year, the combined BOA+HRR module was found to be most suitable (SEN = 86%, SPE = 91%, ACU = 90%). In children aged 1–3 years, SSAT module was found to be suitable (SEN=98%,SPE= 85%, ACU= 91.2%. For the 3–6 years age group, SRT was found suitable (SEN =95%, SEN=??, ACU=91.43%). When test-based screening methods are not feasible, standalone HRR (0–1 year) and PQ (3–6 years) modules remain viable alternatives, meeting the 80% sensitivity and specificity benchmarks to screen for >=60dBHL.

## Background

Hearing screening programs aim to detect permanent hearing loss greater than 40 dB HL at the earliest possible stage ( The Joint Committee on Infant Hearing, 2019). Evidence from developed countries demonstrates that Early Hearing Detection and Intervention (EHDI) programs significantly reduce the age of identification to less than one year, enabling timely amplification and intervention. Children diagnosed and fitted with hearing devices within the first few months of life showed speech, language, and literacy development that aligned with their chronological age ( Neumann *et al.*, 2022). Economic evaluations of universal newborn hearing screening revealed that early intervention substantially decreases future treatment costs, leading to long-term savings for healthcare systems and social welfare programs ( Colgan *et al.*, 2012). Consequently, early identification through hearing screening programs can enhance the efficiency of public health and social support services.

In India, 0.6 to 5 per 1000 individuals are estimated to have congenital hearing loss ( Joshi *et al.*, 2023; Verma *et al.*, 2022). The Government’s welfare schemes (including hearing aids, cochlear implants, special education and habilitation) for persons with hearing impairment are available for those with 60 dB HL or greater hearing loss in the better ear (ADIP scheme, RPWD, 2016). This is similar in several other global southern countries, such as Bangladesh, Pakistan, and Kenya ( Ndegwa *et al.*, 2024), where only higher degrees of hearing loss are eligible for welfare schemes, aid, and appliances with public funds.

Although even mild hearing loss has an impact on optimal participation, learning, and development from a public health perspective of the Global South, as a first step, it is pertinent to at least identify children with hearing loss who can utilize and benefit from the existing government schemes.

In India, Government-initiated programs at the community level have implemented disability screening, including hearing loss screening, using questionnaires and informal measures ( Singh *et al.*, 2015; Varshney, 2016). Although these programmes have the potential to reach rural communities, their outcomes are not optimal. Among other reasons, poor outcomes can be attributed to the use of unvalidated checklists, recurrent costs associated with imported hearing screening equipment, and lack of a digital data interface that results in poor tracking and loss to follow-up ( Galhotra & Sahu, 2019; Gupta *et al.*, 2015). Similar issues are commonly reported in southern countries globally.

To overcome such challenges, mobile health (mHealth)-related services have been explored in a few hearing screening programs ( Swanepoel & Clark, 2019; Yimtae *et al.*, 2018; Yousuf Hussein *et al.*, 2016). Some mHealth applications for hearing screening, such as Ear-trumpet ( Amlani, 2015), SHOEBox, ( Yeung *et al.*, 2013) and Ear Scale, ( Chu *et al.*, 2019) are iOS-based, whereas the Hearing Test ( Renda *et al.*, 2016) and Hear Screen ( Swanepoel *et al.*, 2014) are android-based, but use pure-tone-based applications and are validated predominantly on adults. Currently, only twenty-six applications for hearing screening are available for children, ( Frisby *et al.*, 2022) and none of these innovations are for children below 4 years of age. A few innovations for children include tablet-based automated hearing screening in China for preschool screening programs for children above five years of age. However, these screening apps use pure tones that are appropriate for adults and older children, and are not useful for children below 4 years of age ( Kam *et al.*, 2014; Louw *et al.*, 2017).

To address this gap, an m-health hearing screener (SRESHT hearing screener) was developed for use by grassroots-level workers to screen children for hearing loss at the community level. The SRESHT hearing screener includes a calibrated headphone (Sony WHCH 510) and a speaker (JBL Go 2). These commercial transducers were calibrated according to ANSI standards (ANSI/ASA S3.6-2018)( ANSI S3.6, 2018). The stimulus level for screening was set at 60 dB HL to identify children with a 40% disability (disabling hearing loss) based on the RPWD Act, 2016 ( Government of India, 2016). Age-specific subjective hearing screening modules were: i) Behavioral Observation Audiometry (BOA)-based screening (0 to 1 year of age); ii) Speech Spectrum Awareness Task (SSAT)-based screening (> 1 to 3 years of age); and iii) Speech Recognition Task (SRT)-based screening (> 3 to 6 years of age). In addition,) high-risk register checklist and) parental questionnaire were included for all age groups. The development of the SRESHT hearing screener, including details of output calibration, has been published, ( Ramkumar *et al.*, 2025) and interested readers are directed to this article for details.

The present study describes the validation process and outcomes of the SRESHT hearing screener with age-appropriate screening modules for 60 dB HL of hearing loss among children below 6 years of age against the reference standard test.

## Methodology

This study was approved by the Institutional Ethics Committee (IEC – NI/20/OCT/76/113) of Sri Ramachandra Institute of Higher Education and Research (SRIHER). Written informed consent was obtained from the parents prior to their child’s participation.

### Validation of hearing screener against reference standard test

A purposive convenient sampling method was used to include children in the study. The sample size was estimated using the following equation: $\text{N}=\frac{z^{2}P(100-P)}{{\left(\frac{L*P}{100}\right)}^{2}},$ where N is the number of samples, Z (Confidence Interval) = 1.96, p (sensitivity) =80%, q (specificity) = 100-p, and L (precision) = 18% for 0 to 1 year, 15% for 1 to 3 years, and 3 to 6 years was considered based on both prevalence and feasibility in obtaining samples. The estimated sample size for 0-1 year was 28 samples for 1 -3 years and 3-6 years was 43 samples, in both CWNH (children with normal hearing) and CWHI (children with hearing impairment).

Children were recruited from the audiology clinic at Sri Ramachandra Hospital, maternity hospitals, early intervention centers, pre-schools, and play-schools in and around the Chennai district.

***Eligibility criteria***

Infants and children under 6 years of chronological age with Tamil as their native language were included in the study. Infants and children with other disabilities (e.g., Visual Impairment, Autism Spectrum Disorder, Developmental delay) were excluded from the study.

***Test environment and tester***

The SRESHT hearing screener and reference standard screener (described for each test module in the test procedure) were set up in the quieter room available at each of the data collection sites. The ambient noise monitoring feature on the SRESHT hearing screener allows screening to begin only if the ambient noise is less than 50 dB A. The testing room was free from distractions such as people interactions, play area, and electrical noise. Two audiologists (the first author and one clinical audiologist with a postgraduate degree who was not aware of the study’s objective) were involved in data collection. Henceforth, the first author is referred to as the ‘primary audiologist’ and the other clinical audiologist is referred to as the ‘second audiologist. ’

***Test procedure***

The primary and second audiologists visited the centers on predetermined days and performed the validation procedure. The screening test always started with the SRESHT hearing screener, followed by an age-appropriate reference standard screening test. One of the two audiologists performed hearing screening using the SRESHT hearing screener, whereas the other observed the responses to minimize tester bias.

Validation of BOA Screening module:

The child was seated on a mother/caregiver's lap. The speaker was positioned at a 180 °azimuth (directly behind the child) at 1 foot distance from the infant’s ear level. The SRESHT hearing screener is connected to the speaker via Bluetooth. Stimuli centered at 500 Hz, 1 kHz, 2 kHz, and 4 kHz ( Ramkumar *et al.*, 2025) were presented via the SRESHT hearing screener’s software at 60 dB HL via Bluetooth speakers by the primary audiologist. The behavioral responses were observed and noted by a second audiologist. He/she was blinded to child’s hearing status to minimize bias. The expected behavioral responses were eye widening, eye blink, crying, and awakening from slight sleep for children between 0 and 3 months of age, and searching and head turning for children between 3 months and 1 year of age. The ‘pass’ criteria was automated as response in three out of four frequencies as per community hearing screening guidelines by WHO ( World Health Organization, 2021). To reduce tester bias, SRESHT hearing screening (subjective) was always performed first, followed by AABR screening (objective reference standard). This was performed to reduce tester bias.

The same child then underwent objective reference standard Automated Auditory Brainstem Response screening (AABR) using the Otonova ABR screener by Otodynamics at 45 dB nHL (highest limit of the screener) in natural sleep. If the child was not asleep, the same reference standard was used at a later time on the same day or the following day. Considering that automated ABR is quick, yet cannot be performed at 60 dB HL, the maximum level of 45 dB nHL was used as the reference standard test. Only those with a (?) False negatives (i.e.,AABR refer and SRESHT BOA pass) were re-checked with diagnostic ABR at 60 dB HL/75 dB nHL.

Validation of SSAT screening module

The child was seated on the caregiver’s lap or on a low chair, and the stimuli were presented using the SRESHT hearing screener at 60 dB HL via headphones. The stimuli were animal sounds centered at 500 Hz, 1 kHz, 2 kHz, and 4 kHz ( Ramkumar *et al.*, 2025). Behavioral responses, such as searching for the animal/sound source and imitating the animal sounds, were considered as responses for children between 1 and 2 years. A picture pointing response with two alternate forced choice methods was used for children between 2 two three years.. The pass criterion was automated as a response to three out of four stimuli. The same child was then subjected to reference standard objective automated AABR screening at 45 dB nHL during natural sleep. If the child was not asleep, the same reference standard was used at a later time on the same day or the following day. Considering that automated ABR is quick, yet could not be performed at 60 dB HL, the maximum level of 45 dB nHL was used as the reference standard test. Only those with a (?) False negatives (i.e., AABR refer and SRESHT SSAT pass) had to be re-checked with ABR at 60 dB HL/75 dB nHL.

Validation of SRT screening module:

The child was seated on a chair and Bluetooth headphones were placed. The stimuli (Spondee words) were presented using the SRESHT hearing screener through headphones at 60 dB HL to obtain ear-specific responses. The screener display shows four choice-picture paradigms. The child was instructed to tap the picture on the tablet screen based on the words they heard. Ear-specific responses were obtained. Scores were automatically calculated based on the children’s responses to the four choice picture paradigms. Five out of six correct responses (>80% of psychometric function) were considered pass criteria for each ear separately. The child was then tested using standard/conditioned audiometry (500 Hz–4 kHz) through headphones. The second audiologist conducted the conditioned/standard audiometry screening using the KUDUwave Pro diagnostic audiometer as the reference standard. If the child obtained responses at 60 dB HL in 500-4 kHz test frequencies, then it was considered ‘pass. ’

Validation of HRR screening module

The HRR screening (Supplementary file 1) was administered on all children between 0 and 6 years of age who were included in the. The items appeared on the screen automatically based on the child’s age. Responses included’ yes’ or” no’ for each item. The responses obtained were saved on a backend datasheet.

For HRR, two criteria for pass/refer were considered. For 1 item refer criteria, if any 1 item of the HRR checklist was refer, then the overall result was considered as ‘refer. ’ For 2 items refer criteria, only if a minimum of 2 items of the HRR checklist were refer, then the overall result was considered as ‘refer. ’

To validate the module, the pass/refer results of the HRR module were compared with previously obtained reference standard test results based on the child’s age.

Validation of PQ screening module

PQ (Supplementary file 1) was also administered to all children between 0 and 6 years of age. Questions appeared on the screen based on the child’s age. Responses included’ yes’ or ‘no” for each item. The pass/refer results were calculated based on yes/no responses for each item. The responses obtained for each item in the PQ were saved in the backend datasheet.

Two criteria for passing/referring were considered for the PQ screening module. For 1 item refer criteria, if any 1 question of the PQ questionnaire was refer, then the overall result was considered as ‘refer. ’ For 2 items refer criteria, only if a minimum of 2 questions of the PQ questionnaire were refer, then the overall result was considered as ‘refer. ’

To validate the module, the pass/refer results of the PQ module were compared with the previously obtained reference standard test results based on the child’s age.

### Analysis

The Sensitivity (SEN), specificity (SPE), Positive Predictive Values (PPV), Negative Predictive Values (NPV), and accuracy (ACU), along with the confidence intervals (CI), were calculated for each of the hearing screening modules. The pass and refer responses were coded as 0 and 1, respectively.

Infants and children who received a ‘Pass’ in both SRESHT module at 60 dB HL and reference standard at 45 dB nHL (AABR screening) or 60 dB HL (PTA screening), considered as true negatives. Those who received ‘Refer’ in both the SRESHT module at 60 dB HL and the reference standard at 45 dB nHL (AABR screening) or 60 dB HL (PTA screening) were considered ‘true positives. ’ Those who received ‘Refer’ in ‘SRESHT module’ at 60 dB HL, but ‘Pass’ in reference standard at 45 dB nHL (AABR screening) or 60 dB HL (PTA screening), were considered as false positives. Those with ‘Refer’ results in reference standard at 45 dB nHL (AABR screening) or 60 dB HL (PTA screening) and ‘Pass’ results in the SRESHT module, an ABR was performed at 60 dB HL/75 dB nHL to confirm the false negative response. The pass and refer responses were coded as 0 and 1, respectively. Table 2 and Table 3 list the coding conditions for true positives, true negatives, false positives, and false negatives for each module.

To identify the suitability of the screening modules, the criterion of 80% sensitivity and specificity was considered acceptable ( Heidari *et al.*, 2015; Melo *et al.*, 2022; Monaghan *et al.*, 2021). The McNemar test and Chi-square test were used to test the significance of differences in accuracy, as applicable and are detailed in the results.

## Results

### Validation of hearing screener against reference standard test

A total of 316 children [Male (n) = 147; Female (n) =169 females] were included in this validation study. One hundred and ninety-two children had normal hearing and one hundred and twenty four with moderately severe (> 60 dB HL to 80 dB HL) to profound hearing loss (> 80 dB HL) (CWHL). The children were divided into three groups based on the age-appropriate subjective screening modules ( Table 1). The checklist-based HRR & PQ screening module was administered to parents/caregivers of all 316 children who underwent age-appropriate subjective test-based hearing screening modules.

**Table 1.** Participant details for each screening module.

Screening module	Age Range	Mean age	CWNH	CWHI			Total number screened
				MSHL-SHL	PHL	Total CWHI	
BOA screening	2 days to 12 m	3.4 m	92	11	17	28	120
SSAT screening	14 m to 3 yrs	2.1 yrs	48	12	31	43	91
SRT screening	3.1 to 6 yrs	4.3 yrs	52	19	34	53	105
Total	2 days- 6 yrs	-	192	42	82	124	316

*BOA: Behavioral Observation Audiometry; SSAT: Speech Spectrum Awareness Task; SRT: Speech Recognition Task; CWNH: Children with Normal Hearing; CWHI: Children with Hearing Impairment; PHL: Profound Hearing Loss; MSHL: Moderately Severe Hearing Loss; SHL: Severe Hearing loss; m, months; y, years.

The sensitivity, specificity, PPVs, NPVs, and accuracy with confidence intervals were calculated for each module specific to the age group.

The true positives, true negatives, false positives, and false negatives used for calculating SEN, SPE, PPV, NPV, and ACU are tabulated in Table 2 and Table 3.

**Table 2.** Number of true positives, true negatives, false positives and false negatives for each module.

Age range	Modules	True positive (refer in both tests)	False negatives (pass in index test & refer in reference standard)	True negatives (pass in both tests)	False positives(refer in index test & pass in reference standard)
0 to 1 year	BOA	24	4	81	11
1 to 3 years	SSAT	42	1	41	7
3 to 6 years	SRT	50	3	46	6
0 to 1 year	HRR 1 item (0 to 1)	24	4	80	12
	HRR 2 items (0 to 1)	23	5	80	12
	PQ 1 item ( 0 to 1)	16	12	68	24
	PQ 2 items (0 to 1)	15	13	74	18
1 to 3 years	HRR 1 item (1 to 3)	37	6	25	23
	HRR 2 items (1 to 3)	35	8	28	20
	PQ 1 item (1 to 3)	40	3	30	18
	PQ 2 items (1 to 3)	38	5	32	16
3 to 6 years	HRR 1 item (3 to 6)	46	7	42	10
	HRR 2 items (3 to 6)	42	11	43	9
	Pq 1 item (3 to 6)	48	5	43	9
	PQ 2 items (3 to 6)	44	9	43	9

**Table 3.** Number of true positives, true negatives, false positives and false negatives for each module for age-sub groups based on type of responses from child.

Modules	Age range	True positive (refer in both tests)	False negatives (pass in index test & refer in reference standard)	True negatives (pass in both tests)	False positives (refer in index test & pass in reference standard)
BOA module	0 to 3 months	6	2	39	7
	3 to 12 months	17	3	42	4
SSAT	1 to 2 years	10	0	15	3
	2 to 3 years	32	1	26	4

***Validity for each screening module for hearing losses ≥ 60 dB HL***

The results of the validity of each module is provided in Table 4.

**Table 4.** Overall validity of each hearing screening modules for hearing losses ≥ 60 dB HL.

Modules (age)		SEN(%)	CI (95%)	SPE(%)	CI (95%)	PPV(%)	CI (95%)	NPV(%)	CI (95%)	Accuracy	CI (95%)
BOA (0 to 1) *		85.71	67.33-95.97	88.04	79.61-93.88	68.57	55.12-79.49	95.29	89.07-98.05	87.50	80.22-92.83
SSAT (1 to 3) *		97.67	87.71-99.94	85.42	72.24-93.93	85.71	75.13-92.26	97.62	85.48-99.65	91.21	83.41-96.13
SRT (3 to 6) *		94.62	84.34-98.32	88.46	76.56-95.65	89.29	79.65-94.66	93.88	83.57-97.88	91.43	84.35-96.01
HRR (0 to1)	1 item *	85.71	67.33-95.97	86.96	78.32-93.07	66.67	53.60-77.59	95.24	88.94-98.03	86.67	79.25-92.18
	2 items	82.14	63.11-93.94	86.96	78.32-93.07	65.71	52.38-76.95	94.12	87.81-97.26	85.83	78.29-91.53
HRR (1 to 3)	1 item	86.05	72.07-94.70	52.08	37.19-66.71	61.67	53.91-68.87	80.65	65.40-90.18	68.13	57.53-77.51
	2 items	81.40	66.60-91.61	58.33	43.21-72.39	63.64	54.88-71.58	77.78	64.19-87.24	69.23	58.68-78.49
HRR (3 to 6)	1 item	86.79	74.66-94.5	80.77	67.47-90.37	82.14	72.30-89.02	85.71	74.82-92.38	83.81	75.35-90.28
	2 items	79.25	65.89-89.16	82.6	69.67-91.77	82.35	71.72-89.57	79.63	69.48-87.03	80.95	72.13-87.96
PQ (0 to 1)	1 item	57.14%	37.18-75.54	73.91	63.71-82.52	40.00	29.40-51.62	85.00	78.41-89.84	70.00	60.96-78.02
	2 items	53.57%	33.87-72.49	80.43	70.85-87.97	45.45	32.71-58.82	54.55	79.06-89.56	74.17	65.38-81.72
PQ (1 to 3)	1 item	93.02%	80.84-98.54	62.50	47.35-76.05	68.97	60.45-76.36	90.91	76.66-96.82	76.92	66.91-96.82
	2 items	88.37%	74.92-96.11	66.67	51.59-79.60	70.37	61.08-78.24	86.49	73.27-93.73	76.92	66.91-85.11
PQ (3 to 6)	1 item *	90.57%	79.34-96.87	82.69	69.67-91.77	84.21	74.53-90.67	89.58	78.72-95.24	86.67	78.64-92.51
	2 items	83.02%	70.20-91.93	82.69	69.67-91.77	83.02	72.72-89.97	82.69	72.23-89.77	82.86	74.27-89.51

Note: BOA: Behavioral Observation Audiometry; SSAT: Speech Spectrum Awareness Task; SRT: Speech Recognition Task; HRR: High Risk Register; PQ: Parental Questionnaire; CI: Confidence Interval; SEN: Sensitivity; SPE: Specificity; PPV: Positive Predictive Values; NPV: Negative Predictive Value; ‘*’: Modules shortlisted based on 80% criteria.

The sensitivity and specificity of all three subjective test-based hearing-screening modules (BOA, SSAT, and SRT) fulfilled the benchmark criteria of 80% ( Heidari *et al.*, 2015; Melo *et al.*, 2022)

For the checklist/questionnaire-based modules, refer criteria of one item and two items were considered. In the 0–1 year age group, the HRR module with 1- and 2-item refer criteria fulfilled the benchmark criteria of sensitivity and specificity. The HRR 1-item refer criterion demonstrated higher absolute values of sensitivity, specificity, and accuracy and was therefore selected as the preferred refer criterion for this age range ( Table 4), although the difference was not statistically significant on the McNemar test (p =1.00). The PQ module (one item and two item refer criteria) did not fulfill the benchmark criteria and was not selected for this age group.

For the 1–3 years age group, both the HRR and PQ (1 item and 2 item criteria did not fulfill the benchmark criteria for specificity, although sensitivity values fulfilled the benchmark. Therefore, neither the HRR nor PQ modules were selected for this age group.

For the 3–6 years age group, the PQ module (both 1 item and 2 item refer criteria) and HRR with 1 item criterion fulfilled the benchmark criteria of sensitivity and specificity. However, the PQ 1 item reference criterion demonstrated higher values of sensitivity, specificity, and accuracy and was therefore selected, though statistically insignificant on the McNemar test (p = .125). The McNemar test also showed no significant difference between the HRR 1 item and PQ 1 item (p=1.00). Therefore, HRR 1 item (0–1 year) and PQ 1 item (3–6 years) were selected for each age range.

Since in the BOA and SSAT modules, the type of responses obtained from the child varied based on the age within these modules, a sub-group analysis was performed to determine the validity within the sub-age groups based on the type of response.

Validity of BOA and SSAT based on type of responses within age group

The samples in each module were further sub-grouped based on the type of response to the stimuli, as per age. Table 5 shows the validity of the subgroups based on response type.

**Table 5.** Validity of BOA and SSAT hearing screening modules based on age-sub groups.

	Type of responses	SEN(%)	CI (95%)	SPE(%)	CI (95%)	PPV(%)	CI (95%)	NPV(%)	CI (95%)
**BOA**									
0 to 3m	Eye blink, eye widening, crying/startle/cessation of activity	75.00	55-90	84.68	60-95	46.15	34-79	95.12	78-100
3m - 1y	Head turn, searching for the sound	85.00	66-97	91.30	78-98	80.95	73-89	93.33	78-99
**SSAT**									
1 to 2 years	Imitating the animal sounds, locating/searching for the sound source (behavioral)	100	69-100	83.33	58-96	76.92	54-90	100	78-100
2 to 3 years	Picture pointing task (2AFC)	96.97	82-100	86.67	68-96	88.88	74-94	96.29	78-99

*BOA, behavioral observation audiometry; SSAT, speech spectrum awareness task; SRT, speech recognition task; CI, confidence interval; SEN, sensitivity; SPE, specificity; PPV, positive predictive value; NPV, negative predictive value.

BOA screening module Infants were sub-grouped from 0 to 3 months of age [n= 54; CWNH = 46, CWHI = 8) and from 3 months to 1 year of age (n = 66; CWNH = 46, CWHI = 20) based on the expected reflexive responses to the stimulus ( Table 5). The results indicated that the sensitivity and specificity of infants between 3 months to 1 year fulfilled the benchmark criteria of 80% for both sensitivity and specificity. However, for 0–3 months of age, a benchmark criterion of 80% was not achieved for sensitivity. Hence, the BOA module was valid for the age group of three months to one year.

SSAT screening module: Children were sub-grouped from 1 to 2 years (28; CWNH [n) =18, CWHI [n) =10] and from 2 to 3 years of age (n = 64; CWNH =30, CWHI =33) based on the type of responses from the child. The results indicated that sensitivity and specificity fulfilled the benchmark criteria of 80% for both age subgroups. A chi-square test also revealed no significant difference between the two groups (χ²(1) = 1.500, p = .22). Hence, the overall SSAT module was considered valid across both age groups (1–2 and 2–3 years).

Combination of screening modules for each age group

To assess whether combining subjective and checklist/questionnaire-based screening improved the validity, age-appropriate subjective test modules were combined with the age-specific checklist/questionnaire module selected for each age group. This resulted in a total of two combinations: one for the age group of 0 to 1 year [BOA (subjective test module) + HRR (checklist]) and one for 3 to 6 years of age [SRT (subjective test module) + PQ (questionnaire)] ( Table 6).

**Table 6.** Validity of combination of hearing screening modules.

Age	Combination of modules	SEN(%)	CI (95%)	SPE(%)	CI (95%)	PPV(%)	CI (95%)	NPV(%)	CI (95%)	Accuracy(%)	CI (95%)
0 to 1 year	BOA+ HRR	85.71	67.33- 95.97	91.30	83.58- 96.17	75.00	60.33- 85.54	95.45	86.43- 98.12	90.00	83.18- 94.73
3 to 6 years	SRT + PQ	90.57	79.34- 96.87	88.46	76.56- 95.65	88.89	78.95- 94.46	90.20	79.89- 95.52	89.52	82.03- 94.65

*BOA, behavioral observation audiometry; SRT, speech recognition task; CI, confidence interval; SEN, sensitivity; SPE, specificity; PPV, positive predictive value; NPV, negative predictive value.

For the 0–1 year age group, the standalone BOA module was compared to the combined BOA+HRR module. The absolute values of specificity and accuracy of the BOA+HRR combined module were higher than those of the standalone BOA module, although the difference was not statistically significant in the McNemar test (p = 0.25). Therefore, the BOA+HRR module was selected as the preferred module for this age group.

For the 1–3 years age group, the standalone SSAT module was preferred because no other module fulfilled the benchmark criteria in this age group.

For the 3–6 years age group, the standalone SRT module was compared to the combined SRT+PQ module. The absolute values of the sensitivity and accuracy of the standalone SRT module were higher than those of the SRT+PQ combined module, although the difference was not statistically significant in the McNemar test (p = 0.50). Therefore, the SRT module was selected as the preferred module for this age group.

Subgroup analysis was conducted based on the degree of hearing loss to determine the validity of each module in screening for various degrees of hearing loss.

Degree of hearing loss wise validity of each hearing screening modules

The validity was determined for children with varying degrees of hearing loss categorized by age group ( Table 7 & Table 8). Children were sub-grouped as those with a) moderately severe to severe hearing loss (MS-SHL) (50 dB HL – 80 dB HL) (n=42) and b) profound hearing loss (PHL) (>80 dB HL) (n=82) based on the WHO criteria of hearing loss for each of the screening modules ( Olusanya *et al.*, 2019).

**Table 7.** Degree of hearing loss (moderately severe- severe) wise validity of each hearing screening modules.

Screening module (age in years)	n	MSHL-SHL									
		SEN (%)	CI (95%)	SPE (%)	CI (95%)	PPV (%)	CI (95%)	NPV (%)	CI (95%)	ACCURACY	CI (95%)
BOA(0 to 1)	11	72.73	39.03- 93.98	88.04	79.61- 93.88	42.11	27.28- 58.51	96.43	91.12- 98.61	86.41	78.25- 92.37
SSAT (1 to 3)	12	91.67	61.52- 99.79	85.42	72.24- 93.93	61.11	43.69- 76.09	97.62	86.22- 99.63	86.67	75.41- 94.06
SRT (3 to 6)	19	89.47	66.86- 98.70	88.46	76.56- 95.65	73.91	56.79- 85.93	95.83	86.07- 98.85	88.73	79.00- 95.01
HRR (0 to 1)	11	72.73	39.03- 93.98	88.04	79.61- 93.88	42.11	27.28- 58.51	96.43	91.12- 98.61	86.41	78.25- 92.37
PQ (3 to 6)	19	78.95	54.43- 93.95	82.69	69.67- 91.77	62.50	46.83- 75.93	91.49	81.69- 96.28	81.69	70.73- 89.87
BOA+ HRR (0 to 1)	11	72.73	39.03- 93.98	91.30	83.58- 96.17	50.00	31.98- 68.02	96.55	91.41- 98.66	89.32	81.69- 94.55
SRT+ PQ (3 to 6)	19	78.95	54.43- 93.95	88.46	76.56- 95.65	71.43	53.21- 84.69	92.00	82.72- 96.51	85.92	75.62- 93.03

*n: number of samples; MSHL: moderately severe hearing loss; SHL: severe hearing loss; BOA: behavioral observation audiometry; SSAT: speech spectrum awareness task; SRT: speech recognition task; HRR: high-risk register; PQ: parental questionnaire; CI: confidence interval; SEN: sensitivity; SPE: specificity; PPV: positive predictive value; NPV: negative predictive value.

**Table 8.** Degree of hearing loss (profound hearing loss) wise validity of each hearing screening modules.

Screening module (age in years)	n	PHL									
		SEN (%)	CI (95%)	SPE (%)	CI (95%)	PPV (%)	CI (95%)	NPV (%)	CI (95%)	ACCURACY	CI (95%)
BOA(0 to 1)	17	94.12	71.31- 99.85	88.04	79.61- 93.88	59.26	45.20- 71.95	98.78	92.35- 99.82	88.99	81.56- 94.18
SSAT (1 to 3)	31	100.00	88.78- 100.0	85.42	72.24- 93.93	81.58	69.07- 89.78	100.00	91.40- 100	91.14	82.59- 96.36
SRT (3 to 6)	34	97.06	84.67- 99.93	88.46	76.56- 95.65	84.62	72.11- 92.13	97.87	86.93- 99.69	91.86	83.95- 96.66
HRR (0 to 1)	17	94.12	71.31- 99.85	88.04	79.61- 93.88	59.26	45.20- 71.95	98.78	92.35- 99.82	88.99	81.56- 94.18
PQ (3 to 6)	34	97.06	84.67- 99.93	82.69	69.67- 91.77	78.57	66.87- 86.95	97.73	86.13- 99.67	88.37	79.65- 94.28
BOA + HRR (0 to 1)	17	94.12	71.31- 99.85	91.30	83.58- 96.17	66.67	50.51- 79.67	98.82	92.61- 99.82	91.74	84.90- 96.15
SRT + PQ (3 to 6)	34	97.06	84.67- 99.93	88.46	76.56- 95.65	84.62	72.11- 92.13	97.87	86.93- 99.69	91.86	83.95- 96.66

*n: number of samples; MSHL: moderately severe hearing loss; SHL: severe hearing loss; BOA: behavioral observation audiometry; SSAT: speech spectrum awareness task; SRT: speech recognition task; HRR: high-risk register; PQ: parental questionnaire; CI: confidence interval; SEN: sensitivity; SPE: specificity; PPV: positive predictive value; NPV: negative predictive value.

For moderately severe to severe (MS-SHL) hearing loss category, only the standalone SSAT and SRT modules fulfilled the benchmark criteria of 80% sensitivity and specificity. For the profound degree of hearing loss (PHL) category, all selected standalone and combination modules fulfilled the benchmark criteria of 80% sensitivity and specificity.

The absolute values of sensitivity and accuracy were higher in the PHL subgroups than in the MS-SHL subgroups for BOA, SSAT, SRT, HRR (0–1 year age), PQ (3–6 years), and the combination modules BOA+HRR (0 to 1 year) and SRT+PQ (3 to 6 years). However, the chi-square test showed no significant difference between the MS-SHL and PHL subgroups of all selected modules [BOA (χ²(1) = 2.406, p = .121); SSAT (χ²(1) = 2.583; p = .238), SRT (χ²(1) = 1.532, p = .46); HRR (χ²(1) = 2.406, p = .121), PQ (χ²(1) = 5.227, p = .30); BOA+HRR (χ²(1) = 2.406, p = .281), SRT+PQ (χ²(1) = 5.227, p = .30)].

These findings suggest that the subjective test modules (SSAT and SRT) for 1–3 years of age and 3–6 years of age are valid across degrees of hearing loss, ranging from MS-SHL to PHL. However, the BOA and checklist/questionnaire-based modules, when used independently, are valid for PHL ( Table 7 and Table 8).

**Summary of findings**

The results suggest that the most suitable screening modules to screen >= 60 dB HL were;

For the age-group of **0–1 year,** **BOA+HRR** combined module was selected, as the combined validity (SEN = 86%, SPE = 91% & ACU =90%) was better than that of the standalone BOA (SEN= 86%, SPE = 88% & ACU = 87.5%) screening module by 3 % for specificity and 3% for accuracy.

For the age group of **1–3 years**, **SSAT** alone (SEN= 98%, SPE = 85% & ACU=91.2%) was selected, as the other modules did not fulfill the benchmark criteria of 80%.

For the age group of **3–6 years**, **SRT** alone (SEN = 95%, SPE = 89% & ACU = 91.43%) was selected, as the combined validity of SRT with PQ screening module (SEN = 91%, SPE = 89% & ACU=89.5%) did not yield better sensitivity rather it reduced the sensitivity by 4% and accuracy by 2%.

When screening using BOA, SSAT, or SRT is not feasible, HRR (0–1year) and PQ (3–6 years) as a standalone screening tool may still be used as they fulfill the 80% benchmark criteria of sensitivity and specificity.

## Discussion

The findings from this study suggest that the sensitivity of the SRESHT hearing screener ranges from 86 to 98% and specificity ranges from 85 to 90% across the three subjective test-based modules. The accuracy of similar mHealth hearing screening tools was determined using sensitivity and specificity ( Kam *et al.*, 2014; Wu *et al.*, 2014; Yimtae *et al.*, 2018).

The SRESHT hearing screener is an affordable screener with age-appropriate subjective hearing screening modules for children aged 0–6 years. Among the available mHealth-based hearing applications, only a few are designed specifically for children who are above or older than 4 years of age ( Dillon *et al.*, 2018; Khoza-Shangase & Kassner, 2013; Yeung *et al.*, 2013; Yimtae *et al.*, 2018). A few of these smart phone hearing screeners, such as the Kids hearing game ( Xiao *et al.*, 2020) and P.E.T.I.T hearing screeners, ( Samelli *et al.*, 2017) are tested in children aged 3 years and are not found to be suitable for these children due to the complexity of the task involved ( Chu *et al.*, 2019; Samelli *et al.*, 2017; Wu *et al.*, 2014). However, the SRESHT hearing screener consists of interesting yet simple tasks, making it suitable for young children. The SRESHT hearing screener is currently designed to screen for 'moderately severe' and above degrees of hearing loss according to the WHO revised classification of hearing loss ( Olusanya *et al.*, 2019).

Although the guidelines for NHS programs in various countries emphasize permanent hearing loss of 40 dB HL as a target population, our study aimed at 60 dB HL as an initial step. This level was prioritized as i) welfare scheme criteria in India are for hearing losses ≥ 60 dB HL in the better ear, and ii) to establish a sustainable model of hearing screening by incorporating an affordable validated hearing screener to the existing public sector system that is likely to apply to other global South countries as well. Sustainability remains inconsistent in many hearing-screening programs for LMICs ( Neumann *et al.*, 2019). Hence, the current study was undertaken initially with the country-specific requirement of identifying disabling hearing loss and task shifting to grassroots workers.

It is noteworthy that the current study findings of the BOA screening module overcame the aforementioned challenges and were accurate enough to screen for hearing losses greater than 60 dB HL (40%). For 3 months to 1 year of age, the sensitivity and specificity were comparable to those of other smartphone-based hearing screeners ( Melo *et al.*, 2022) and objective screening measures such as OAE ( Heidari *et al.*, 2015), but were lower for neonates. This is possibly due to the lack of overt responses in neonates, who are often in a deeper sleep state. The reason for high sensitivity and specificity could be the appropriate procedure followed in the hearing screening by blinding the observer, thereby avoiding observer bias on responses. For the youngest age group (0–1 year), noise makers are known to be robust, ( Ramesh *et al.*, 2012; Valencia, 2015) which was reconfirmed by Ramkumar *et al.* (2025) ( Ramkumar *et al.*, 2025). Earlier research on behavioral hearing screening, such as auditory response cradle and crib-o-gram in infants, was known to be effective in identifying severe to profound hearing loss but missed milder hearing losses. However, these screening methods are not suitable for mass-screening programs ( Hayes, 2003).

The SSAT screening module for children between 1 and 3 years of age was accurate, with a sensitivity of 97% and specificity of 85%. This can be attributed to two factors. First, in early childhood, animal sounds are intriguing ( Nolte *et al.*, 2016) and suitable for audiometric screening/testing. Nolte *et al.* (2016) found that the frequency-specific animal sound test (FAST) with animal sounds of different frequencies was valid as a diagnostic test for children above 2.5 years of age. Second, region-specific animal sounds were chosen, which could have contributed to children’s familiarity with the stimuli, thereby improving the appropriate response. The third reason could be the simple response mode, which requires only a limited attention span from the child.

In this age group of 1 to 3 years, warble/ pure tones or objective measures were commonly used and were reported to be more effective and reliable comparable to the subjective tests ( Kanji & Khoza-Shangase, 2016). To our knowledge, no attempts have been made to develop or validate mHealth-based subjective hearing screeners for children younger than three years of age. However, our findings suggest that using suitable age-appropriate stimuli and response methods can result in an optimum sensitivity and specificity for hearing loss of ≥ 40 %.

The SRT module for 3 to 6 year olds with spondee words and picture paradigm response mode was found to be appropriate for this age group, with accuracy comparable to other studies that used tablet-based screening tests for older children ( Mahomed-Asmail *et al.*, 2016; Samelli *et al.*, 2020; Yimtae *et al.*, 2018). Even with a psychometric function of 80%, the specificity (90%) and sensitivity (95%) of measures obtained to detect 40% hearing loss were high. Pure tones and automated audiometry have been used in screening tests for preschool-aged children in some mHealth applications ( Kam *et al.*, 2014; Wu *et al.*, 2014; Yimtae *et al.*, 2018). In the P.E.T.I.T application, the response mode was two choice yes/no options, and the sensitivity and specificity were reported to be 85% and 97%, respectively, for children between 6 and 12 years of age ( Samelli *et al.*, 2020). Sound scouts, ( Dillon *et al.*, 2018) a game-based screening for children above 4 years of age, were reported to have a sensitivity of 85% and specificity of 98% for hearing losses above 30 dB HL. Similarly, PASS versions 1 and 2 ( Yimtae *et al.*, 2018) with Thai words and a picture-based response paradigm were validated at 4 to 5 years of age with sensitivity and specificity of 90% and 93%, respectively. Hence, it can be stated that the SRT module for 3 to 6 years has similar sensitivity and specificity for children from 3 to 6 years of age as any of the similar mHealth tools developed for children worldwide.

Overall, the subjective test-based screening modules of the SRESHT hearing screener are valid and appropriate for age groups as young as 3 months of age, whereas the available validated smart phone-based applications using pure-tones/speech for children were found to be accurate for children only above four years of age ( De Swanepoel *et al.*, 2014; Dillon *et al.*, 2018; Yeung *et al.*, 2013). In addition, many of these mHealth apps have been developed with a rationale to use them for school entry level screening and not for those who are younger ( Dillon *et al.*, 2018). It is also noteworthy that the age range of 0–3 years has not been considered in any of the reported innovations so far.

In general, studies using unvalidated HRR as part of case studies ( Khoza-Shangase & Harbinson, 2015; Lee *et al.*, 2016) in a few studies explored an unvalidated HRR checklist as a standalone screening tool ( Khan *et al.*, 2018; Olusanya, 2011) and found that it was simple, quick, and could be easily administered by nurses or community health workers. However, the use of an appropriate context-specific high-risk register for hearing loss is crucial ( Khan *et al.*, 2018). Our study findings showed the highest sensitivity of the HRR checklist (1 item refers to criteria) for infants below 1 year of age, which could be due to the) inclusion of context-specific items in the checklist; in contrast to paper-based systems, cloud-based data management ( Jayawardena *et al.*, 2019) allows for appropriate data management and the ability to obtain individual item findings as needed.

According to the findings of this study, for children above 3 years of age, a parental questionnaire including questions on audition, progressive late-onset hearing loss, and middle ear diseases is a quick and useful screening tool for hearing loss greater than 60 dB HL. Many school-based screening programs in developing countries include a questionnaire as part of the case history and audiological test battery, but they are often not validated. Only a few studies have validated and used it as a standalone tool ( Coninx *et al.*, 2009; Schaefer *et al.*, 2019). However, the number of items in these questionnaires is more than 20, which requires a longer time to administer, making them less efficient. In contrast, in the current study, the number of items in the questionnaire for each age range was between five and seven. Also the use of ‘app-based digitized HRR/PQ screening module’ for screening facilitates automatization according to the selected age range and facilitates data management

### Limitations

This validated hearing screener may overlook hearing losses of less than 60 dB HL, as the current study validated the SRESHT hearing screener for children with disabling hearing losses (>60 dB HL) as defined by the RPWD Act (2016). This was primarily to strengthen the current Indian scenario, with a validated low-cost standardized hearing screening method for greater triaging of children who are likely to benefit from social welfare schemes at the grassroots level. Since it is intended for use by community-level grassroots workers, the tool was also validated when the intended end-user performed the screening. These results will be published in a separate publication.

### Future studies

Currently, the SRESHT hearing screener has been validated for 60 dB HL and above degrees of hearing losses. A field validation of the tool was undertaken to determine the agreement in results between screening performed using the tool by a grassroots worker versus an audiologist. Validation was also initiated to lower the screening level to 40 dB HL to accommodate and screen for mild hearing loss.

## Data Availability

The datasets generated and analyzed during this study is available at Open Science Framework: Validation of an m-health based hearing screener (SRESHT) for children below 6 years of age in Southern India. https://doi.org/10.17605/OSF.IO/A97EC. ( Joshi *et al.*, 2026). This project contains the following underlying data: Data set final validation. (Data on Hearing screening outcomes). Supplementary file. (Appendix 1 to 6). Data is available under the terms of the CC-By Attribution 4.0 International.
