# Supplementary material for: VALIDATION OF A BEHAVIOURAL, SPEECH SPECTRUM/SPEECH BASED mHEALTH HEARING SCREENER FOR CHILDREN
Source: Wellcome Open Res. 2026 May 5;11:107. Originally published 2026 Feb 12. [Version 2] doi: 10.12688/wellcomeopenres.25773.2 (PMC13179490; doi:10.12688/wellcomeopenres.25773.2)
Supplement: Peer review history

# Reviewer report: Reviewer response for version 2

**DOI:** 10.21956/wellcomeopenres.29214.r154870

**Published:** 2026-05-15

**Authors:** Archana Gundmi

**Affiliations:** 1 Department of Speech and Hearing, Manipal College of Health Professions, Manipal Academy of Higher Education (MAHE), Manipal, Karnataka, India

While the revised manuscript shows clear improvement, a few **methodological ambiguities and minor presentation issues** still remain. There is some lack of clarity in the **false-negative validation pathway**, particularly due to ambiguous wording (e.g., “(?) false negatives”), making it unclear whether all false-negative cases were confirmed using diagnostic ABR or only a subset; this has implications for the accuracy of sensitivity estimates and reproducibility. Additionally, although the authors have attempted to justify the use of **45 dB nHL AABR as a reference standard for a 60 dB HL screening target**, the explanation could be made more explicit to ensure readers clearly understand the rationale and any potential for misclassification bias. The handling of **borderline or inconsistent responses** is also not clearly described, which may affect clinical interpretation. Furthermore, the **HRR and PQ scoring procedures**, while explained, would benefit from clearer structuring (e.g., a table or flowchart) to enhance replicability.

Is the work clearly and accurately presented and does it cite the current literature?

Partly

If applicable, is the statistical analysis and its interpretation appropriate?

Yes

Are all the source data underlying the results available to ensure full reproducibility?

Yes

Is the study design appropriate and is the work technically sound?

Yes

Are the conclusions drawn adequately supported by the results?

Yes

Are sufficient details of methods and analysis provided to allow replication by others?

Partly

Reviewer Expertise:

Rehabilitative audiology,

I confirm that I have read this submission and believe that I have an appropriate level of expertise to confirm that it is of an acceptable scientific standard.

# Reviewer report: Reviewer response for version 2

**DOI:** 10.21956/wellcomeopenres.29214.r154871

**Published:** 2026-05-13

**Authors:** Kumar Seluakumaran

**Affiliations:** 1 University of Malaya, Kuala Lumpur, Malaysia

The authors have responded constructively to the reviewer comments and have made meaningful revisions that address the major methodological concerns raised in the initial review. The explanations regarding reference standards, sampling strategy, and study limitations are satisfactory.

 A few minor issues remain:

1. The calibration description (currently provided in paragraphs 7–9 of the Background section) would be more appropriately placed in the Methods section, as this is methodological information rather than background context.
2. There is some repetition in the justification for the use of ABR. Currently, both the *Validation BOA* and *SSAT screening module* sections contain essentially the same explanatory text beginning with *“Considering that automatic ABR is quick...”*. This could be consolidated to improve readability and avoid redundancy.
3. Minor grammatical and editorial corrections are still needed, as a few language errors remain in the revised manuscript.

Overall, I am satisfied that the substantive concerns have been addressed, and I do not believe further detailed review is necessary.

Is the work clearly and accurately presented and does it cite the current literature?

Yes

If applicable, is the statistical analysis and its interpretation appropriate?

Partly

Are all the source data underlying the results available to ensure full reproducibility?

Yes

Is the study design appropriate and is the work technically sound?

Yes

Are the conclusions drawn adequately supported by the results?

Partly

Are sufficient details of methods and analysis provided to allow replication by others?

Partly

Reviewer Expertise:

Hearing loss, Hearing screening test, auditory psychophysics

I confirm that I have read this submission and believe that I have an appropriate level of expertise to confirm that it is of an acceptable scientific standard.

# Reviewer report: Reviewer response for version 1

**DOI:** 10.21956/wellcomeopenres.28374.r152660

**Published:** 2026-04-09

**Authors:** Archana Gundmi

**Affiliations:** 1 Department of Speech and Hearing, Manipal College of Health Professions, Manipal Academy of Higher Education (MAHE), Manipal, Karnataka, India

This study presents the development and validation of the SRESHT mHealth-based hearing screener designed for children aged 0–6 years in resource-constrained settings. The tool incorporates age-specific subjective screening modules—Behavioral Observation Audiometry (BOA), Speech Spectrum Awareness Task (SSAT), and Speech Recognition Task (SRT)—along with checklist-based modules (High-Risk Register [HRR] and Parental Questionnaire [PQ]).

 The validation was conducted on 316 children using age-appropriate reference standards (AABR and conditioned audiometry). The study reports that:

- BOA + HRR is optimal for 0–1 year
- SSAT alone for 1–3 years
- SRT alone for 3–6 years

All selected modules met ≥80% sensitivity and specificity criteria for detecting ≥60 dB HL hearing loss. The study highlights the feasibility of deploying an affordable, scalable screening solution for low-resource settings.

**1. Is the work clearly and accurately presented, and does it cite the current literature?**

**Answer: Partly**

**Strengths**

- The manuscript is comprehensive and logically structured.
- Literature cited is relevant and includes recent work (up to 2024–2025).
- Strong justification for focusing on ≥60 dB HL in the Global South context.

**Concerns & Suggestions**

- **Minor inconsistencies and typographical errors**:
  - Grammar issues throughout (e.g., “children between 2 two three years”)
- **Abstract requires refinement**:
  - Missing specificity value for SRT
  - Formatting inconsistencies (percentages, spacing)
- Some **sentences are overly long and difficult to follow**, especially in Background and Discussion.

**2. Is the study design appropriate and is the work technically sound?**

**Answer: Yes**

**Strengths**

- Appropriate **diagnostic validation design**
- Use of **age-specific screening paradigms** is a major strength
- Blinding between audiologists reduces bias
- Use of **reference standards (AABR and PTA)** is appropriate

**Minor Concerns**

- Use of **45 dB nHL AABR as reference for 60 dB HL screening** needs clearer justification
- Convenience sampling may limit generalizability

**Suggestions**

- Add a brief **justification for reference level mismatch**
- Mention **potential selection bias** explicitly in limitations

**3. Are sufficient details of methods and analysis provided to allow replication?**

**Answer: Partly**

**Strengths**

- Detailed description of:
  - Screening procedures
  - Stimuli and response criteria
  - Pass/refer logic
- Inclusion of calibration standards (ANSI)

**Concerns**

- Some procedural details unclear:
  - Exact **criteria for false negative confirmation**
- HRR and PQ scoring logic could be better structured

**4. Is the statistical analysis and its interpretation appropriate?**

**Answer: Yes**

**Strengths**

- Appropriate use of:
  - Sensitivity, specificity, PPV, NPV, accuracy
  - Confidence intervals
  - McNemar and Chi-square tests
- Clear benchmark criterion (≥80%)

**Minor Suggestions**

- Clarify **handling of borderline cases**
- Brief explanation of **clinical relevance of PPV/NPV in low-resource settings**

**5. Are all the source data underlying the results available?**

**Answer: Yes**

**Strengths**

- Data availability via **Open Science Framework**
- Transparency enhances reproducibility

**6. Are the conclusions adequately supported by the results?**

**Answer: Yes**

**Strengths**

- Conclusions align with reported findings
- Appropriate emphasis on:
  - Age-specific screening
  - Feasibility in LMICs
- Discussion acknowledges limitations

**Minor Suggestions**

- Avoid **overgeneralization beyond ≥60 dB HL**
- Strengthen statement on **future validation for 40 dB HL**

**Major Issues to be Addressed (Essential for Scientific Soundness)**

1. **Abstract corrections**
  - Add missing specificity values (SRT)
2. **Methodological clarity**
  - Clarify ABR validation pathway for false negatives
3. **Language and formatting**
  - Extensive proofreading required
4. **Consistency in reporting**
  - Ensure all values (SEN, SPE, ACU) match across text and tables

**Minor Issues (Recommended Improvements)**

- Add **flowchart of study procedure**
- Improve readability of long paragraphs
- Standardize abbreviations at first use
- Enhance clarity in subgroup analysis description

**Overall Recommendation**

 The study is **methodologically strong, clinically relevant, and highly valuable**, especially for low-resource settings. However, **clarity, consistency, and presentation issues must be addressed** before indexing.

Is the work clearly and accurately presented and does it cite the current literature?

Partly

If applicable, is the statistical analysis and its interpretation appropriate?

Yes

Are all the source data underlying the results available to ensure full reproducibility?

Yes

Is the study design appropriate and is the work technically sound?

Yes

Are the conclusions drawn adequately supported by the results?

Yes

Are sufficient details of methods and analysis provided to allow replication by others?

Partly

Reviewer Expertise:

Rehabilitative audiology,

I confirm that I have read this submission and believe that I have an appropriate level of expertise to confirm that it is of an acceptable scientific standard, however I have significant reservations, as outlined above.

# Response

**Published:** 2026-04-19

**Authors:** Vidya Ramkumar

**Affiliations:** Sri Ramachandra Institute of Higher Education and Research (Deemed to be University), Chennai, Tamil Nadu, India

We thank the reviewer for reviewing the article and for their valuable comments to improve the work. We also thank the reviewer for mentioning the strengths of the study as well as concerns and areas that needs improvement and clarity. We have taken all that concerns into considerations and clarified them in the manuscript. **1.Concerns & Suggestions Minor inconsistencies and typographical errors: Grammar issues throughout (e.g., “children between 2 two three years”) ** Abstract requires refinement: Missing specificity value for SRT Formatting inconsistencies (percentages, spacing) Some sentences are overly long and difficult to follow, especially in Background and Discussion **Response:** Abstract has been refined. Missing value has been added to the abstract. Typographical errors have been corrected throughout the manuscript. **2. Is the study design appropriate and is the work technically sound? Answer: Yes** **Strengths ** Appropriate diagnostic validation design Use of age-specific screening paradigms is a major strength Blinding between audiologists reduces bias Use of reference standards (AABR and PTA) is appropriate Minor Concerns Use of 45 dB nHL AABR as reference for 60 dB HL screening needs clearer justification Convenience sampling may limit generalizability Suggestions Add a brief justification for reference level mismatch Mention potential selection bias explicitly in limitations **Response:** A brief justification has been added under the procedure for validation of each module in the method section. A statement regarding exclusion of other disabilities and selection bias has been added in the limitation. **3. Are sufficient details of methods and analysis provided to allow replication? Answer: Partly ** Strengths Detailed description of: Screening procedures  Stimuli and response criteria  Pass/refer logic   Inclusion of calibration standards (ANSI) Concerns Some procedural details unclear: Exact criteria for false negative confirmation HRR and PQ scoring logic could be better structured **Response:** Detailed ABR was conducted to confirm the false negative responses (i.e., Pass in SRESHT hearing screener and Refer in ABR screening/PTA screening). **4. Is the statistical analysis and its interpretation appropriate? ** Answer: Yes Strengths Appropriate use of: Sensitivity, specificity, PPV, NPV, accuracy Confidence intervals McNemar and Chi-square tests Clear benchmark criterion (≥80%) Minor Suggestions Clarify handling of borderline cases Brief explanation of clinical relevance of PPV/NPV in low-resource settings **Response:** Brief explanation of PPV/NPV in community setting has been added under the limitation in the discussion section. **5. Are all the source data underlying the results available? Answer: Yes ** Strengths Data availability via Open Science Framework Transparency enhances reproducibility **6. Are the conclusions adequately supported by the results? Answer: Yes ** Strengths Conclusions align with reported findings Appropriate emphasis on: Age-specific screening Feasibility in LMICs Discussion acknowledges limitations **Minor Suggestions ** Avoid overgeneralization beyond ≥60 dB HL Strengthen statement on future validation for 40 dB HL **Response:** A statement on future validation and field validation has been added in the discussion section **Major Issues to be Addressed (Essential for Scientific Soundness) **

1. Abstract corrections

Add missing specificity values (SRT)

1. Methodological clarity

Clarify ABR validation pathway for false negatives

1. Language and formatting

 Extensive proofreading required

1. Consistency in reporting

 Ensure all values (SEN, SPE, ACU) match across text and tables **Response:** Abstract has been refined and missing values has been added. A brief description of detailed ABR for false negatives has been mentioned in the procedure. Consistency, language and formatting has been verified. **Minor Issues (Recommended Improvements) ** Add flowchart of study procedure Improve readability of long paragraphs Standardize abbreviations at first use Enhance clarity in subgroup analysis description **Response:** We have made the necessary revision in the manuscript to include all the possible changes. **Overall Recommendation ** The study is methodologically strong, clinically relevant, and highly valuable, especially for low-resource settings. However, clarity, consistency, and presentation issues must be addressed before indexing. **Response:** We thank the reviewer for their valuable time and effort in reviewing our manuscript and providing constructive comments to improve the overall clarity of the manuscript.

# Reviewer report: Reviewer response for version 1

**DOI:** 10.21956/wellcomeopenres.28374.r148003

**Published:** 2026-03-10

**Authors:** Kumar Seluakumaran

**Affiliations:** 1 University of Malaya, Kuala Lumpur, Malaysia

The manuscript presents the validation of the SRESHT mHealth-based hearing screener for children aged 0–6 years. The study addresses an important gap in early hearing screening tools that are feasible for low- and middle-income countries and builds on the authors’ earlier work describing the development of the SRESHT screener (Ramkumar et al 2025). (Ref 1)

 The topic is highly relevant, and the study includes a substantial dataset collected across multiple age groups. Overall, this work represents a valuable contribution toward improving accessible hearing screening solutions in resource-limited settings. However, several methodological clarifications and conceptual issues would benefit from further explanation before the manuscript can be considered for indexing.

 1. The SRESHT screener is designed to detect hearing loss ≥60 dB HL, whereas the reference standard for infants and young children is automated ABR screening conducted at 45 dB nHL. Because ABR thresholds are expressed in dB nHL rather than behavioural dB HL, the relationship between these levels is not clearly explained. Providing additional clarification on how these measures correspond would help readers better interpret the reported validity estimates. In addition, the manuscript states that only false negatives (AABR refer and SRESHT pass) were rechecked using diagnostic ABR at a higher level (comparable to 60 dB HL / 75 dB nHL). It would be helpful if the authors could clarify whether diagnostic confirmation was applied consistently across all outcome categories.

 If confirmatory testing was performed only for a subset of outcomes, this may introduce potential verification bias, as some groups receive additional diagnostic confirmation while others do not. For example, if 45 dB nHL corresponds approximately to behavioural thresholds of ~30–40 dB HL, children with thresholds between ~40–60 dB HL could fail the reference standard but either pass or fail the screening target depending on the screener’s performance. In such cases, some positive cases could potentially be misclassified. Discussing how this may influence the validity estimates would strengthen the methodological transparency of the study.

 2. The manuscript states that the aim was to identify the optimal screening module for detecting hearing loss ≥60 dB HL across age groups. However, the study design appears to pre-assign specific screening modules to each age group (BOA for 0–1 year, SSAT for 1–3 years, and SRT for 3–6 years), and each module is validated only within its assigned group. Therefore, the study appears to primarily evaluate the validity of predefined age-specific modules, rather than comparing multiple screening approaches within the same age group to determine which performs best. The authors may wish to clarify this distinction and consider slightly reframing the study objective and conclusions to better reflect the study design.

 3. Different reference standards were used across age groups (AABR screening at 45 dB nHL for children aged 0–3 years and Conditioned or standard audiometry at 60 dB HL for children aged 3–6 years).These approaches assess hearing using different physiological and behavioural paradigms. As a result, the validity estimates across age groups may not be directly comparable. A brief explanation of the rationale for selecting different reference standards for each age group would help readers better understand the methodological choices and their implications for interpreting screening performance.

 4. Participants were recruited using purposive convenience sampling from clinical and intervention settings. The study sample includes 124 children with hearing impairment among 316 participants (~39%), which is considerably higher than the prevalence typically observed in community-based screening populations. While this approach is understandable for validation studies, it would be helpful if the authors could clarify the sampling strategy and discuss how this higher prevalence may influence estimates such as predictive values and the generalizability of the results to community screening settings.

 5. The study aims to detect hearing loss ≥60 dB HL. However, the subgroup analysis defines moderately severe to severe hearing loss as 50–80 dB HL, which includes thresholds below the stated screening target. In addition, since screening tools are typically designed to detect the presence of clinically significant hearing loss rather than differentiate severity levels, the authors may wish to briefly explain the rationale for subdividing hearing loss severity in the screening analysis. Also, the finding that sensitivity was higher for profound hearing loss than for moderately severe hearing loss is expected for screening tests, since children with profound loss are unlikely to respond to stimuli at the screening level. A brief acknowledgment of this point in the discussion may help contextualize the subgroup analysis.

 6. The manuscript reports validity estimates for combinations of screening modules (e.g., BOA + HRR and SRT + PQ), but it is not clear how the results of the two tests were combined to determine the final pass/refer classification. Clarifying whether the combination followed a parallel rule (refer if either test refers), a serial rule (refer only if both tests refer),or another decision algorithm would improve the clarity and reproducibility of the analysis.

 7. The manuscript indicates that the headphones and external speaker were calibrated according to ANSI standards. While some information is provided in the earlier development publication, it would be helpful to briefly summarize the calibration steps in the present manuscript, both for headphone and wireless speaker. Based on ANSI, free-field speaker output levels are typically measured using a mannequin or real-ear measurement to account for ear canal and head-related amplification, and acoustic characteristics of the test environment are usually controlled. I presume these steps were not taken. If simplified calibration procedures were used for screening purposes, this should be sufficient, but I suggest that authors should describe these methods explicitly rather than stating compliance with ANSI standards.

 8. The manuscript provides a formula for sample size estimation but does not cite a reference. The formula presented in the paper appears to differ from commonly used methods. For diagnostic accuracy studies estimating sensitivity and specificity, established approaches are described in Buderer (1996) (Ref 2). Diagnostic accuracy sample size calculations typically incorporate expected prevalence, desired confidence interval width, and anticipated sensitivity and specificity. It would therefore be helpful if the authors could clarify the formula used, define all parameters, and provide an appropriate methodological reference.

 9. Several tables (particularly Tables 2–8) contain numerous statistical measures (TP, TN, FP, FN, sensitivity, specificity, PPV, NPV, accuracy, and confidence intervals), which makes them somewhat difficult to read. The tables could potentially be simplified by presenting the key metrics (e.g., sensitivity, specificity, and accuracy) in the main manuscript while moving additional statistics to supplementary material. In addition, most results are currently presented only in tables. Including graphical representations such as bar charts comparing sensitivity and specificity across BOA, SSAT, and SRT modules may make the findings easier to interpret. A simple flowchart illustrating the screening pathway for each age group (BOA + HRR for 0–1 years, SSAT for 1–3 years, and SRT for 3–6 years) may also improve clarity, but this is optional.

 10.The discussion could be further strengthened by comparing this behavioural screening approach with other low-cost smartphone-based OAE screening technologies, which have been explored in community screening programs (Ali et al 2025; Chan et al 2022). (Ref 3, 4)

**11.**The SSAT procedure appears to involve picture identification of animal sounds. This task may resemble a speech identification or discrimination task rather than a pure awareness task. The terminology could be clarified.

 12.The abstract appears to contain an incomplete result:

*“SRT (SEN =95%, SEN=??, ACU=91.43%)”*

 The specificity value appears to be missing. In addition, references are typically avoided in abstracts.

 13.Testing was conducted in the “quieter room available” with ambient noise below 50 dB A. It would be helpful to clarify whether ambient noise levels were recorded across sites and whether environmental variability was assessed. In addition, it appears that the smartphone’s internal microphone was used for ambient noise monitoring, although this is not explicitly acknowledged both in this and the preceding paper. Clarifying whether this approach was validated in the earlier development study would be helpful.

 14. Explaining why screening set at 60 dB HL helps to identify children with a 40% disability (disabling hearing loss) in the discussion will be helpful. Is this related to functional communication ability being reduced by roughly 40%? How is this percentage derived?

 15. Children with other disabilities were excluded from the study. Since such children may also undergo hearing screening in real-world settings and may have higher rates of hearing loss, acknowledging this as a study limitation may be helpful.

 16.Minor grammatical issues are present throughout the paper. The manuscript would benefit from careful proofreading.

Is the work clearly and accurately presented and does it cite the current literature?

Yes

If applicable, is the statistical analysis and its interpretation appropriate?

Partly

Are all the source data underlying the results available to ensure full reproducibility?

Yes

Is the study design appropriate and is the work technically sound?

Yes

Are the conclusions drawn adequately supported by the results?

Partly

Are sufficient details of methods and analysis provided to allow replication by others?

Partly

Reviewer Expertise:

Hearing loss, Hearing screening test, auditory psychophysics

I confirm that I have read this submission and believe that I have an appropriate level of expertise to confirm that it is of an acceptable scientific standard, however I have significant reservations, as outlined above.

## References

[1] : Development and Beta Validation of an mHealth-Based Hearing Screener (SRESHT) for Young Children in Resource-Limited Countries: Pilot Validation Study. *JMIR Formative Research* .2025;9: 10.2196/53460 10.2196/53460 PMC1177328239805111

[2] : Statistical Methodology: I. Incorporating the Prevalence of Disease into the Sample Size Calculation for Sensitivity and Specificity. *Academic Emergency Medicine* .1996;3(9) : 10.1111/j.1553-2712.1996.tb03538.x 895-900 10.1111/j.1553-2712.1996.tb03538.x 8870764

[3] : An Open-Source Smartphone Otoacoustic Emissions Test for Infants. *Pediatrics* .2025;155(3) : 10.1542/peds.2024-068068 10.1542/peds.2024-068068 39909065

[4] : An off-the-shelf otoacoustic-emission probe for hearing screening via a smartphone. *Nature Biomedical Engineering* .2022;6(11) : 10.1038/s41551-022-00947-6 1203-1213 10.1038/s41551-022-00947-6 PMC971752536316369

# Response

**Published:** 2026-04-19

**Authors:** Vidya Ramkumar

**Affiliations:** Sri Ramachandra Institute of Higher Education and Research (Deemed to be University), Chennai, Tamil Nadu, India

We thank the reviewer for his positive feedback and critical comments to bring more clarity to the manuscript. **Response to comments are below**

**The manuscript presents the validation of the SRESHT mHealth-based hearing screener for children aged 0–6 years. The study addresses an important gap in early hearing screening tools that are feasible for low- and middle-income countries and builds on the authors’ earlier work describing the development of the SRESHT screener (Ramkumar et al 2025). (Ref 1) The topic is highly relevant, and the study includes a substantial dataset collected across multiple age groups. Overall, this work represents a valuable contribution toward improving accessible hearing screening solutions in resource-limited settings. However, several methodological clarifications and conceptual issues would benefit from further explanation before the manuscript can be considered for indexing.**

1. **The SRESHT screener is designed to detect hearing loss ≥60 dB HL, whereas the reference standard for infants and young children is automated ABR screening conducted at 45 dB nHL. Because ABR thresholds are expressed in dB nHL rather than behavioural dB HL, the relationship between these levels is not clearly explained. Providing additional clarification on how these measures correspond would help readers better interpret the reported validity estimates. In addition, the manuscript states that only false negatives (AABR refer and SRESHT pass) were rechecked using diagnostic ABR at a higher level (comparable to 60 dB HL / 75 dB nHL). It would be helpful if the authors could clarify whether diagnostic confirmation was applied consistently across all outcome categories. If confirmatory testing was performed only for a subset of outcomes, this may introduce potential verification bias, as some groups receive additional diagnostic confirmation while others do not. For example, if 45 dB nHL corresponds approximately to behavioural thresholds of ~30–40 dB HL, children with thresholds between ~40–60 dB HL could fail the reference standard but either pass or fail the screening target depending on the screener’s performance. In such cases, some positive cases could potentially be misclassified. Discussing how this may influence the validity estimates would strengthen the methodological transparency of the study.**

  Response: We thank the reviewer for this valuable comment. In this validation study, the SRESHT screener was compared against age-appropriate automated ABR conducted at 45 dB nHL, which represents the maximum screening limit of the screening equipment that was used as the reference standard. A child was expected to pass both SRESHT (60 dB HL) and AABR (45 dB nHL) if hearing was normal. Children with SRESHT pass and ABR screening refer were classified as false negatives, while SRESHT refer with ABR screening pass were considered false positives, with AABR screening serving as the reference standard. Diagnostic ABR (BERA) was performed in all cases with ABR screening refer (including false negatives and true positives cases). However, available prior diagnostic ABR reports were reviewed and cross-verified wherever applicable. Diagnostic BERA was not conducted in cases that passed ABR screening, as these were considered normal. This clarification has now been incorporated under the “Test Procedure” section    

**2. The manuscript states that the aim was to identify the optimal screening module for detecting hearing loss ≥60 dB HL across age groups. However, the study design appears to pre-assign specific screening modules to each age group (BOA for 0‒1 year, SSAT for 1‒3 years, and SRT for 3‒6 years), and each module is validated only within its assigned group. Therefore, the study appears to primarily evaluate the validity of predefined age-specific modules, rather than comparing multiple screening approaches within the same age group to determine which performs best. The authors may wish to clarify this distinction and consider slightly reframing the study objective and conclusions to better reflect the study design**.   Response: Yes, the pre-assigned screening modules were tested on children at each age group. However, along with the age-specific screening modules, questionnaires (HRR and PQ) specific to each age group were also validated for each age group. Hence, the aim was mentioned as ‘to identify the optimal screening module (age specific module or the age specific questionnaire or the combination of both) for detecting hearing loss >= 60 dB HL. This has been reframed for clarification in the abstract.  

**3. Different reference standards were used across age groups (AABR screening at 45 dB nHL for children aged 0–3 years and Conditioned or standard audiometry at 60 dB HL for children aged 3–6 years). These approaches assess hearing using different physiological and behavioural paradigms. As a result, the validity estimates across age groups may not be directly comparable. A brief explanation of the rationale for selecting different reference standards for each age group would help readers better understand the methodological choices and their implications for interpreting screening performance. ** Response: The validity estimates across age groups were not directly compared. The rationale for selecting different reference standards for each group is given below.   For children between 0 to 3 years of age, auditory brainstem response (ABR) screening at 45 dB nHL was the screening test as there was no other standardized behavioral screening test was available for this age group. Hence, a physiological screening test was used. For children between 3 and 6 years of age, pure tone audiometry screening was standardized and widely used as a screening tool. Hence for the age group of 3 and 6 years of age, CPA/PTA screening was used as the reference standard. This is now explained briefly in the test procedure 1 ^st^ paragraph under methods section.  

**4. Participants were recruited using purposive convenience sampling from clinical and intervention settings. The study sample includes 124 children with hearing impairment among 316 participants (~39%), which is considerably higher than the prevalence typically observed in community-based screening populations. While this approach is understandable for validation studies, it would be helpful if the authors could clarify the sampling strategy and discuss how this higher prevalence may influence estimates such as predictive values and the generalizability of the results to community screening settings. ** Thanks for the comment and a brief explanation has been added in the discussion section under the sub heading limitations “While the results showed high values for validity estimates”    

**5. The study aims to detect hearing loss ≥60 dB HL. However, the subgroup analysis defines moderately severe to severe hearing loss as 50‒80 dB HL, which includes thresholds below the stated screening target. In addition, since screening tools are typically designed to detect the presence of clinically significant hearing loss rather than differentiate severity levels, the authors may wish to briefly explain the rationale for subdividing hearing loss severity in the screening analysis. Also, the finding that sensitivity was higher for profound hearing loss than for moderately severe hearing loss is expected for screening tests, since children with profound loss are unlikely to respond to stimuli at the screening level. A brief acknowledgment of this point in the discussion may help contextualize the subgroup analysis. **   Response: A brief explanation for including subgroup analysis and the suggested point has been added in the 9 ^th^ paragraph of discussion starting as ‘The subgroup analysis based on’.  

**6. The manuscript reports validity estimates for combinations of screening modules (e.g., BOA + HRR and SRT + PQ), but it is not clear how the results of the two tests were combined to determine the final pass/refer classification. Clarifying whether the combination followed a parallel rule (refer if either test refers), a serial rule (refer only if both tests refer), or another decision algorithm would improve the clarity and reproducibility of the analysis**.   Response: Decision algorithm for combination of screening modules included a ‘serial rule’ in which hearing screening was considered as ‘refer’ only if both tests are refer. This table with decision rule for all the modules had been now added in the supplementary material for better clarity.    

**7. The manuscript indicates that the headphones and external speaker were calibrated according to ANSI standards. While some information is provided in the earlier development publication, it would be helpful to briefly summarize the calibration steps in the present manuscript both for headphone and wireless speaker. Based on ANSI, free-field speaker output levels are typically measured using a mannequin or real-ear measurement to account for ear canal and head-related amplification, and acoustic characteristics of the test environment are usually controlled. I presume these steps were not taken. If simplified calibration procedures were used for screening purposes, this should be sufficient, but I suggest that authors should describe these methods explicitly rather than stating compliance with ANSI standards. ** Response: Thank you for the comment. Appropriate calibration for both the transducers were carried out and the procedure is now added to the

**8. The free field output speakers levels were calibrated using the standard method where a standardized reference point was chosen specifically where the center of the subject’s head would be during testing. . A volunteer was seated in a chair and the ear level was marked from the floor. The sound level meter was placed vertically so the microphone of the SLM was at the reference point. A free field type microphone was used at a 0 degree angle for sound field calibration. Each stimuli was presented with center frequencies of 500 and 4 and the corresponding dB SPL were measured. The SLM reading in dB SPL was added with the RETSPL values for sound field testing at 0 degree azimuth as in ANSI S3.6 2010.    **   The wireless headphones were calibrated using the wilber’s method where the The RETSPL of the commercial bluetooth headphones were derived initially. Mean thresholds of 10 normal hearing individuals measured using TDH39 headphones in a standard audiometer (Harp Inventis). Then threshold of the same individuals in volume levels (%) measured using SRESHT hearing screener hardware with commercial headphones. Both were compared and RETSPL was derived using Wilber method as described in Guo et al. (2021).  

**9. The manuscript provides a formula for sample size estimation but does not cite a reference. The formula presented in the paper appears to differ from commonly used methods. For diagnostic accuracy studies estimating sensitivity and specificity, established approaches are described in Buderer (1996) (Ref 2). Diagnostic accuracy sample size calculations typically incorporate expected prevalence, desired confidence interval width, and anticipated sensitivity and specificity. It would therefore be helpful if the authors could clarify the formula used, define all parameters, and provide an appropriate methodological reference. **   The formula used for sample size calculation is n =   where n is the number of samples, Z (Confidence Interval) = 1.96, p (sensitivity) =80%, q (specificity) = 100-p, and L ( relative precision). This formula was used for estimating the sample size with relative precision as described by Lwanga and Lameshow (1991). The citation has been added in the manuscript now.   Citation: Lwanga, Stephen Kaggwa, Lemeshow, Stanley & World Health Organization (1991). Sample size determination in health studies : a practical manual World Health Organization. https://iris.who.int/handle/10665/40062  

**10.Several tables (particularly Tables 2–8) contain numerous statistical measures (TP, TN, FP, FN, sensitivity, specificity, PPV, NPV, accuracy, and confidence intervals), which makes them somewhat difficult to read. The tables could potentially be simplified by presenting the key metrics (e.g., sensitivity, specificity, and accuracy) in the main manuscript while moving additional statistics to supplementary material. In addition, most results are currently presented only in tables. Including graphical representations such as bar charts comparing sensitivity and specificity across BOA, SSAT, and SRT modules may make the findings easier to interpret. A simple flowchart illustrating the screening pathway for each age group (BOA + HRR for 0–1 years, SSAT for 1–3 years, and SRT for 3–6 years) may also improve clarity, but this is optional. **   Response: As per the suggestion, the PPVs and NPVs and their confidence intervals were moved to supplementary material 2. This improved clarity of the keymetrics in the main manuscript. The graphical representation were not added because the information from the table would be repeated in the graphical form. **11.The discussion could be further strengthened by comparing this behavioural screening approach with other low-cost smartphone-based OAE screening technologies, which have been explored in community screening programs (Ali et al 2025; Chan et al 2022). (Ref 3, 4) **   Response: A line comparing the BOA screening with recent smartphone-based innovations in objective OAE screening has been added to discussion. **12.The SSAT procedure appears to involve picture identification of animal sounds. This task may resemble a speech identification or discrimination task rather than a pure awareness task. The terminology could be clarified. ** As explained in the method section under the sub heading ‘Validation of SSAT screening module’, “Behavioral responses, such as searching for the animal/sound source and imitating the animal sounds, were considered as responses for children between 1 and 2 years. A picture pointing response with two alternate forced choice methods was used for children between 2 two three years”. Hence, the terminology was chosen SSAT to include not only the discrimination reponse but also the awareness response. **13.The abstract appears to contain an incomplete result: “SRT (SEN =95%, SEN=??, ACU=91.43%)” The specificity value appears to be missing. In addition, references are typically avoided in abstracts.** This was an incomplete and typological error. The changes in the abstract has now been added as   “SRT (SEN =95%, SPE = 89%, ACU=91.43%)” 15. Testing was conducted in the “quieter room available” with ambient noise below 50 dB A. It would be helpful to clarify whether ambient noise levels were recorded across sites and whether environmental variability was assessed. In addition, it appears that the smartphone’s internal microphone was used for ambient noise monitoring, although this is not explicitly acknowledged both in this and the preceding paper. Clarifying whether this approach was validated in the earlier development study would be helpful. **Response:** The ambient noise were recorded at different sites (eg: class room of Anganwadi preschools, kitchen and storage room of Anganwadi preschools, classroom without students and closed windows in play schools, classroom away from play area in play schools, in hospital set up, room away from waiting area)  before selecting the most suitable quieter room. Smartphone’s internal microphone was used for ambient noise monitoring however the readings were initially cross checked with the reading of sound level meter and the output measurement was verified in the earlier developmental study. This has now been mentioned in the “test environment and tester sub- section” under the methods section **14. Explaining why screening set at 60 dB HL helps to identify children with a 40% disability (disabling hearing loss) in the discussion will be helpful. Is this related to functional communication ability being reduced by roughly 40%? How is this percentage derived?** **Response:** As per the Rights of Persons with Disabilities Act (2016), individuals with ≥40% hearing disability are classified under benchmark disability and are eligible for government benefits and support service. The formula for disability percentage is Disability (%)= (5× Better Ear puretone average)+Poorer Ear puretone average / ​6. The 40% disability criterion defined under the RPwD Act is derived from a binaural weighting formula. It does not directly correspond to a 40% reduction in hearing or communication ability. However, a screening level of 60 dB HL, effectively identifies individuals with moderately severe to severe hearing loss, who are likely to fall at or above this benchmark disability and experience significant functional communication difficulties. A point has been added to the third paragraph of discussion. **15. Children with other disabilities were excluded from the study. Since such children may also undergo hearing screening in real-world settings and may have higher rates of hearing loss, acknowledging this as a study limitation may be helpful.** **Response:** Thank you for the comment. Children with other disabilities were excluded from the study, because it might act as a confounding bias that affect the clinical validity of the study. However, these children are likely to refer the hearing screening in real world setting. Hence, a field validation in real world setting is also under way as next part of the study. It has now been acknowledged in the limitation section. **16.Minor grammatical issues are present throughout the paper. The manuscript would benefit from careful proofreading.** The manuscript has been proofread and the corrections were made.
